# Prevention of Puerperal Fever

**Published:** 1905-09

**Authors:** 


### Prevention of Puerperal Fever.

B. Crede (*Centralblatt für Gynäkologie,* February 11, 1905,) thinks that it is just as important to sterilise the internal genitals after childbirth as it is to instil the prophylactic silver solution into the eyes of the newly born child. Conditions, of course, are much simpler in the case of the child's eyes, but the principle of preventing development of lurking germs is the same. He regards Zweifel's idea to evacuate all blood clots as a very fortunate one. Extensive experience has proved that collargol is perfectly harmless for the purpose and is a powerful antiseptic and catalytic which kills or attenuates the germs in the uterus or vagina. If systematically used, it would reduce greatly the number of deaths from puerperal fever. Collargol is introduced in the form of a suppository pushed high into the vagina or cervix or into the uterine cavity after delivery, the opening into the vagina then being loosely tamponed with gauze. The formula for this collargol vaginal ball is:

℞ Collargol

Talc pulv ........................................ āā gr. 15.

Olei cacao ........................................ dr. 4½.

M. ft.—Globuli No. x.

Suppository and gauze can be renewed at need. The collargol penetrates into every crevice, sterilises the secretions without injuring the living cells and, to some extent, is absorbed. Even if infection be already present, it is attenuated, but in this case more energetic measures are necessary. The author irrigates with a 1 to 2000 or 5000 collargol solution, introducing a suppository to act during the intervals, with other measures as indicated, using as a last resource intravenous injections of 8 to 10 cubic centimeters of a 2 per cent. collargol solution.—*Monthly Cyclopedia of Practical Medicine,* April, 1905. p. 180.

